# Lipiodol: from intrusion until exile from the tumor microenvironment

**DOI:** 10.18632/oncoscience.584

**Published:** 2023-08-17

**Authors:** Sheena Anand, Jean-François Geschwind, Vahid Etezadi, Nariman Nezami

**Keywords:** lipiodol, deposition, washout, embolization, liver tumor

Lipiodol or ethiodized oil, which is extracted from poppyseed oil, is a transparent straw-colored oil which sinks when combined with water [1]. Lipiodol does not dissolve when combined with aqueous solutions and can be iodinated with I^131^, thus making the compound both imageable and therapeutic. The radiopacity of lipiodol originates from its iodine content.

Discovered in 1901 by Marcel Guerbet, lipiodol became one of the first contrast agents used in radiology in the 1920s and has since mostly been used for hysterosalpingography [2].

The first utilization of lipiodol for conventional transarterial chemoembolization (cTACE) dates to the 1980s in Japan where it was combined with chemotherapeutic agents to treat patients with hepatocellular carcinoma (HCC) because lipiodol was found to be cleared more slowly from cancer cells than from the healthy liver parenchyma, thereby allowing the emulsion of chemotherapy-lipiodol to remain for a longer period of time within the HCC tumor [3]. Since HCC derives its blood supply from the hepatic arteries, these arteries can be catheterized under imaging guidance, after which lipiodol is delivered precisely into the HCC under direct visualization. Lipiodol plays a significant role in cTACE as it acts as a liquid embolization agent, a drug carrier through the emulsion it forms with the chemotherapeutic agents, and finally as a tumor tracking or seeking agent since it is visible under X-ray guidance, and because lipiodol literally seeks out cancer cells. The most commonly used chemotherapy agent used in the USA is doxorubicin followed by cisplatin [4]. Experimental animal models showed the optimum dose of lipiodol to be in the range of 0.1–0.3 mL/kg for super selective cTACE [5] with an upper threshold of approximately 10–15 mL due to the risk of nontarget embolization to the lungs. Throughout the world, cTACE has become one of the most widely used therapies for unresectable HCC [6]. In fact, it has been included in all treatment guidelines for HCC, especially patients with Barcelona Clinic Liver Cancer (BCLC) stage B disease [5]. Although not as commonly used for HCC, cTACE is also used in patients with liver metastasis from colorectal cancer, breast cancer, and neuroendocrine tumors as well as patients with intrahepatic cholangiocarcinoma [6].

Despite widespread diagnostic and therapeutic application of lipiodol, the mechanism of its prolonged retention in hepatic tumors is not well understood [7]. Both primary and metastatic liver tumors largely depend on the hepatic artery to supply nutrients for energy production, growth and spread beyond the liver. Such reliance on the arterial supply is mediated by a number of factors including vascular endothelial growth factor and increased tumor neo-vascularity [8]. Lipiodol exploits neo-vascularity at the level of the tumor microenvironment whereby the plasma membranes of cancer cells are biomedically modified, making them more lipophilic to favor the retention of lipiodol within cancer cells [9].

Because lipiodol is radio-opaque, it can be visualized during the actual delivery/treatment as well as after cTACE is completed to ensure that the entire tumor burden was treated as the bright stain of lipiodol within the tumor readily can be visualized. Previously this was performed with an unenhanced CT scan the day after the procedure before patient discharge but this largely has been replaced by using cone beam CT (CBCT) with a flat panel detector immediately after the procedure, alleviating the need for a full dedicated CT scan [10]. The increased use of CBCT is not only logistically superior because patients do not need to be transported to the CT scanner, but it also provides both volumetric information while the patient is undergoing the procedure as well as real time information [11].

As previously noted, direct visualization of lipiodol after the procedure is critically important to ensure that the entire tumor burden was treated but also because the pattern of distribution and retention of lipiodol within the tumor can be used as excellent prognostic factors of tumor response and patient survival [12]. Indeed, when incomplete lipiodol retention after the first cTACE was observed, it resulted in a high risk of local recurrence [11, 13].

The wash-out of lipiodol from cancer cells is similarly important to understand. Generally, lipiodol is eliminated from cancer cells slowly because of insufficient portal vascularization, and the absence of both reticuloendothelial cells and lymphatic vessels that would clear lipiodol much more readily [14]. Recent retrospective studies of primary and metastatic liver cancer treated with cTACE using 3D quantitative volumetric analysis demonstrated that lipiodol washout is a time-dependent and negative exponential process, with faster washout in patients with colorectal metastasis than those with neuroendocrine tumors. In this study, responders to cTACE had slower lipiodol washout, which could reflect successful embolization of the tumor and slower blood flow to the tumor area and therefor slower washout, or it could represent different vascularity patterns in the responders. In conclusion, this study proposed that that the size of the tumor, enhancing tumor burden at baseline, and lipiodol washout rate could predict the outcome of cTACE in patients with metastatic liver tumors.
